# Thyroglossal Duct Cyst Papillary Carcinoma: A Rare Disease Entity

**DOI:** 10.7759/cureus.70999

**Published:** 2024-10-07

**Authors:** Fayez A Alrohaimi, Sultan M Alqahtani, Abdulaziz B Almutairi

**Affiliations:** 1 Otolaryngology-Head and Neck Surgery, Prince Sultan Military Medical City, Riyadh, SAU; 2 General Practice, King Saud Hospital, Unaizah, SAU

**Keywords:** sistrunk procedure, thyroglossal cyst, thyroglossal duct cyst malignancies, thyroid papillary cancer, total thyroidectomy

## Abstract

A thyroglossal duct cyst is the most common congenital cyst in the cervical region. Thyroid papillary carcinoma incidence in thyroglossal duct cysts is considered to be low. In most cases, the diagnosis of thyroglossal duct cyst papillary carcinoma is made postoperatively. We present a 62-year-old male with thyroid papillary carcinoma which developed from a thyroglossal duct cyst. This was confirmed in a pathologic study after operation. In our case, there was neither lymph node involvement nor invasion of adjacent tissue. The patient then underwent total thyroidectomy along with the Sistrunk operation. Later on, the patient was given adjuvant treatment with radioactive iodide and thyroid suppression therapy. The patient was followed up for one year without any metastasis or recurrence.

## Introduction

The thyroid gland begins its descent from the foramen cecum to a position below the thyroid cartilage during early gestation. As it moves, it leaves behind an epithelial pathway known as the thyroglossal tract, which typically disappears between the 5th and 10th weeks of gestation. If this tract remains incomplete or retains epithelial tissue, it can lead to the formation of a thyroglossal duct cyst. Such remnants can manifest as a fistula, cyst, duct, tract, or even as ectopic thyroid tissue within a duct or cyst. Incomplete closure of the thyroglossal duct increases the likelihood of developing a thyroglossal duct cyst [1]. The thyroglossal duct cyst is regarded as the most common developmental anomaly of the thyroid gland. It accounts for about 7% of midline masses diagnosed in adults and approximately 70% of midline masses in children [2]. Thyroid carcinomas arising from a thyroglossal duct cyst account for just 1% of cases [3]. Most individuals with thyroglossal duct cysts are asymptomatic, typically presenting at a median age of about 40 years. Ucherman in 1915 and Brentano in 1911 are acknowledged as early pioneers in describing neoplasms associated with thyroglossal duct remnants [4]. Definitive surgical management generally requires the excision of both the cyst and its associated tracts and branches. Because of the close connection between the hyoid bone and the tract, the central portion of the hyoid bone is typically removed during this procedure, known as the Sistrunk procedure, to ensure complete removal of the tract. Recurrence is uncommon, except in cases with overlying skin involvement or intraoperative cyst rupture. The need for simultaneous thyroid gland removal in cases of papillary carcinoma associated with the thyroglossal duct is still a matter of debate [3]. Thyroidectomy is advised in cases where there is an enlarged lymph node, a history of neck irradiation, or if the thyroid gland is found to be nodular [5].

## Case presentation

**Figure 1:**
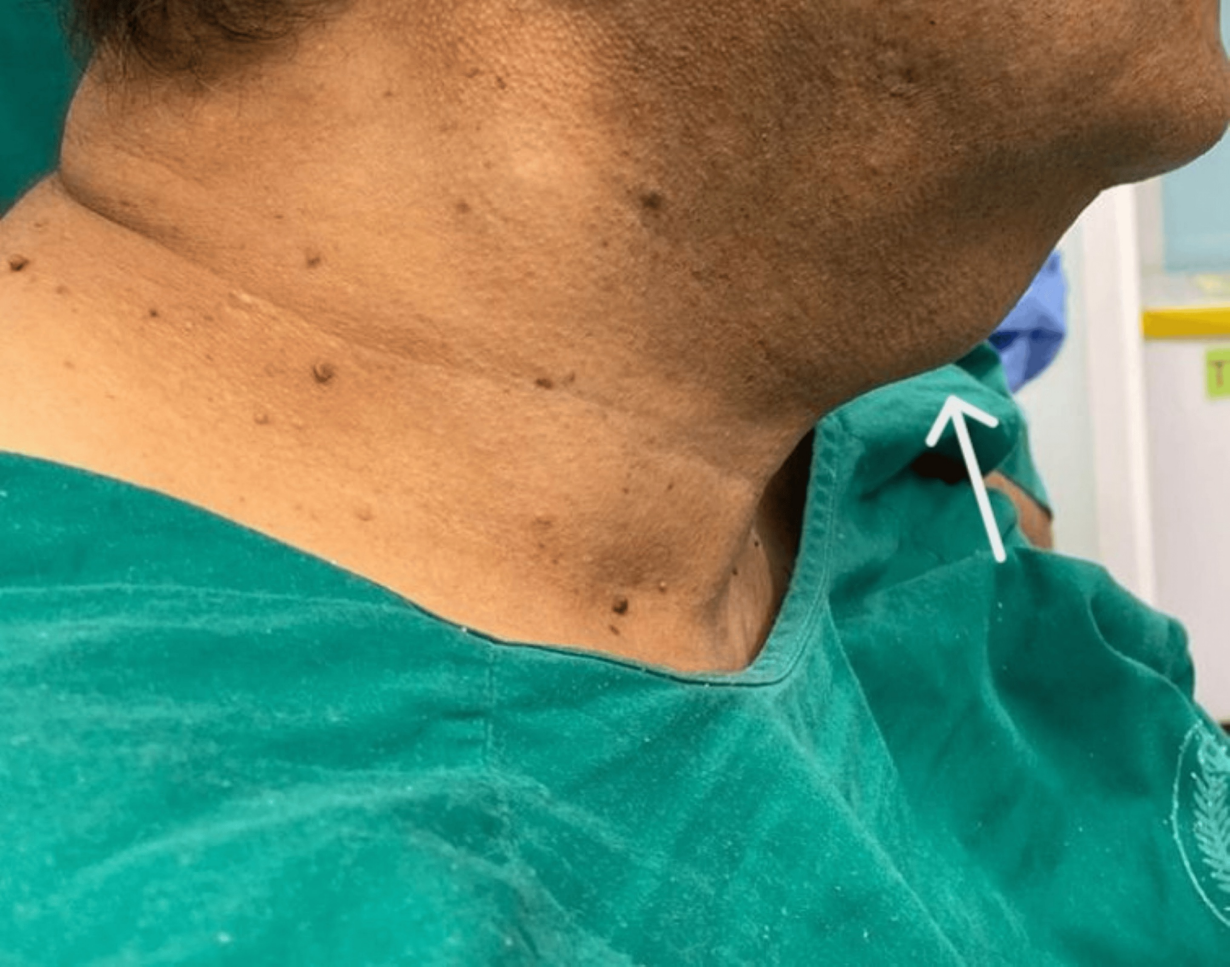
A preoperative image displays an upper midline neck swelling that is painless, slightly mobile, and firm. Measuring about 4 cm by 4 cm, the mass is not fixed to the skin and exhibits no fluctuations. There are no palpable lymph nodes or skin ulcers, and the rest of the head and neck evaluation appears normal.

A 62-year-old male known case of diabetes mellitus, hypertension, ischemic heart disease, and cardiomyopathy was referred to the clinic to assess a painless anterior neck mass. The mass is located in the upper midline of the neck and is associated with dysphagia and odynophagia. The swelling was first noticed two years ago when it was small, but it has gradually increased in size over the past nine months. He reported no history of pain, skin discoloration, numbness or difficulty breathing, weight loss, choking, hoarseness, sleep disturbance, night sweats, fever, history of malignancy, or symptoms of thyroid disease. Physical examination revealed upper midline neck swelling that is not tender, slightly mobile, and firm and did not adhere to the skin and without any fluctuations. The swelling is around 4cm by 4cm in size. There were no palpable lymph nodes and no skin ulcers, and the remainder of the head and neck examination revealed no significant findings (Figure 1).

**Figure 2:**
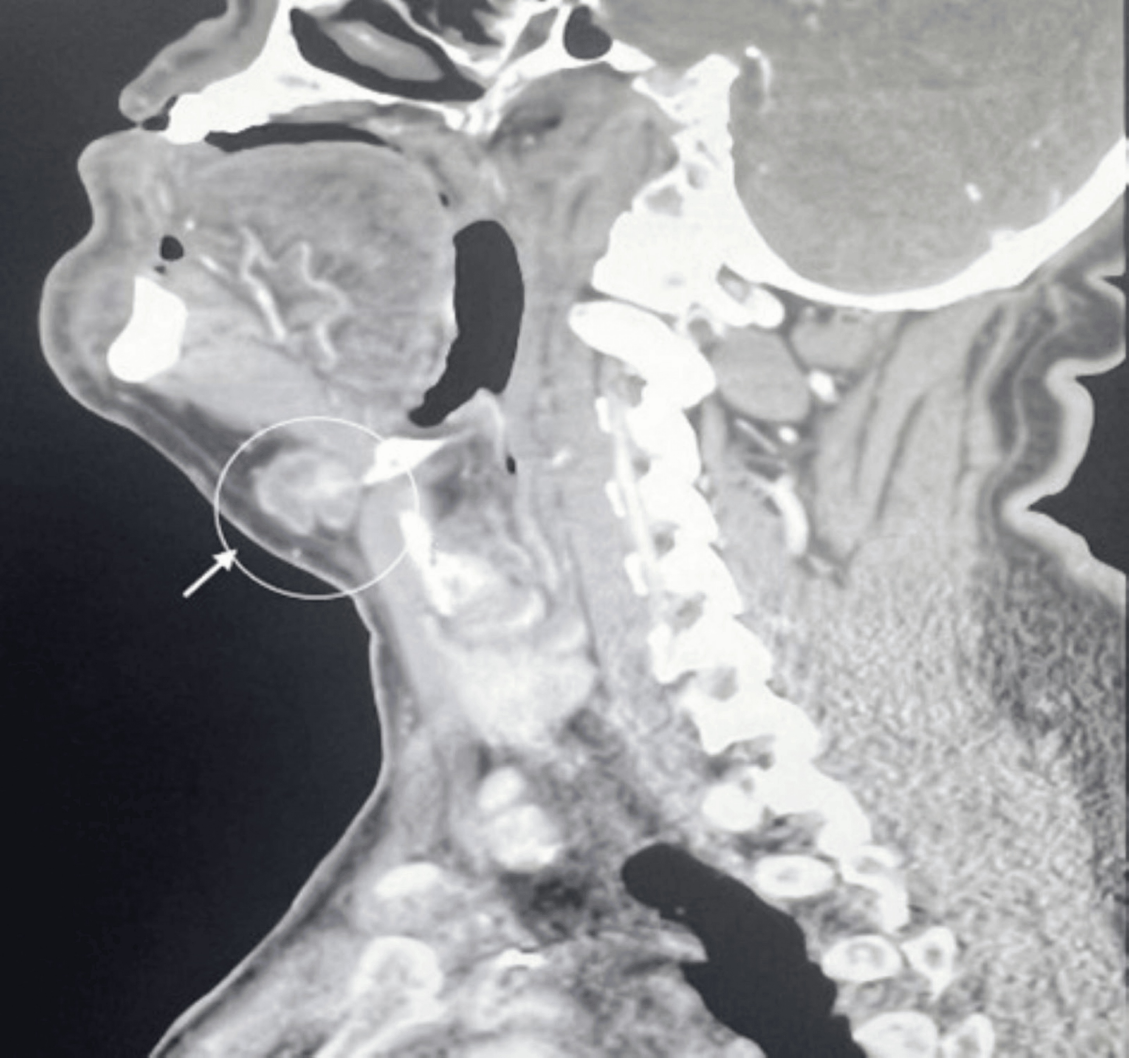
A contrast-enhanced sagittal CT scan of the hyoid bone level shows a multilocular cystic lesion in the midline of the neck, measuring 4 cm by 3 cm. The mass features a thin rim of contrast and contains fluid with a density that exerts pressure on the airway.

**Figure 3:**
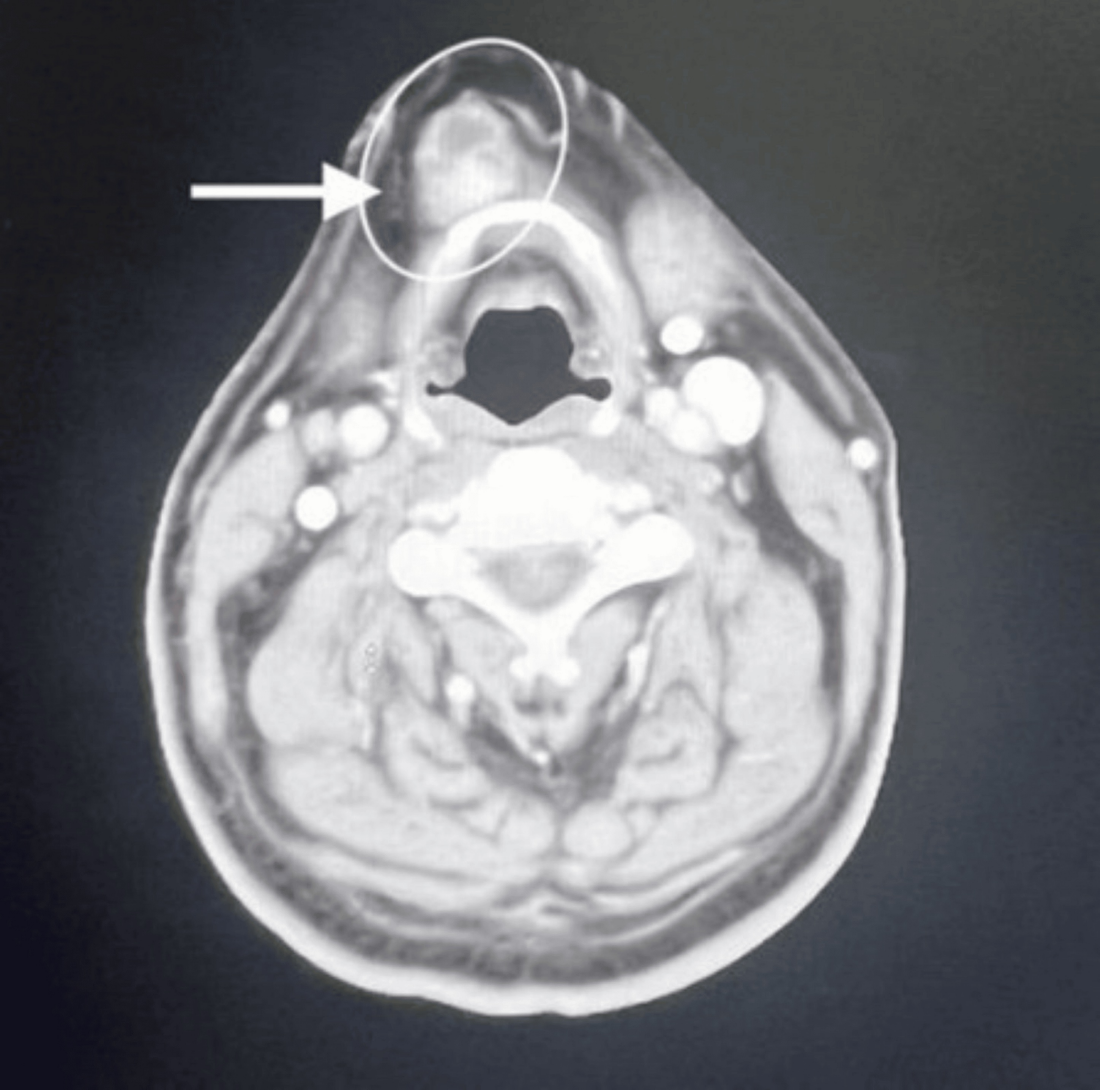
A contrast-enhanced axial CT scan at the hyoid bone level shows a multilocular cystic mass located in the midline of the neck, causing pressure on the airway. There is no evidence of regional lymphadenopathy, and the neck blood vessels are clear.

A contrast-enhanced CT scan at the level of the hyoid bone reveals a multilocular cystic lesion in the anterior midline of the neck, measuring 4 cm by 3 cm. The mass features a thin rim of contrast and contains fluid with a density that creates pressure on the airway. No regional lymphadenopathy and neck vasculatures are patent (Figures 2, 3).

**Figure 4:**
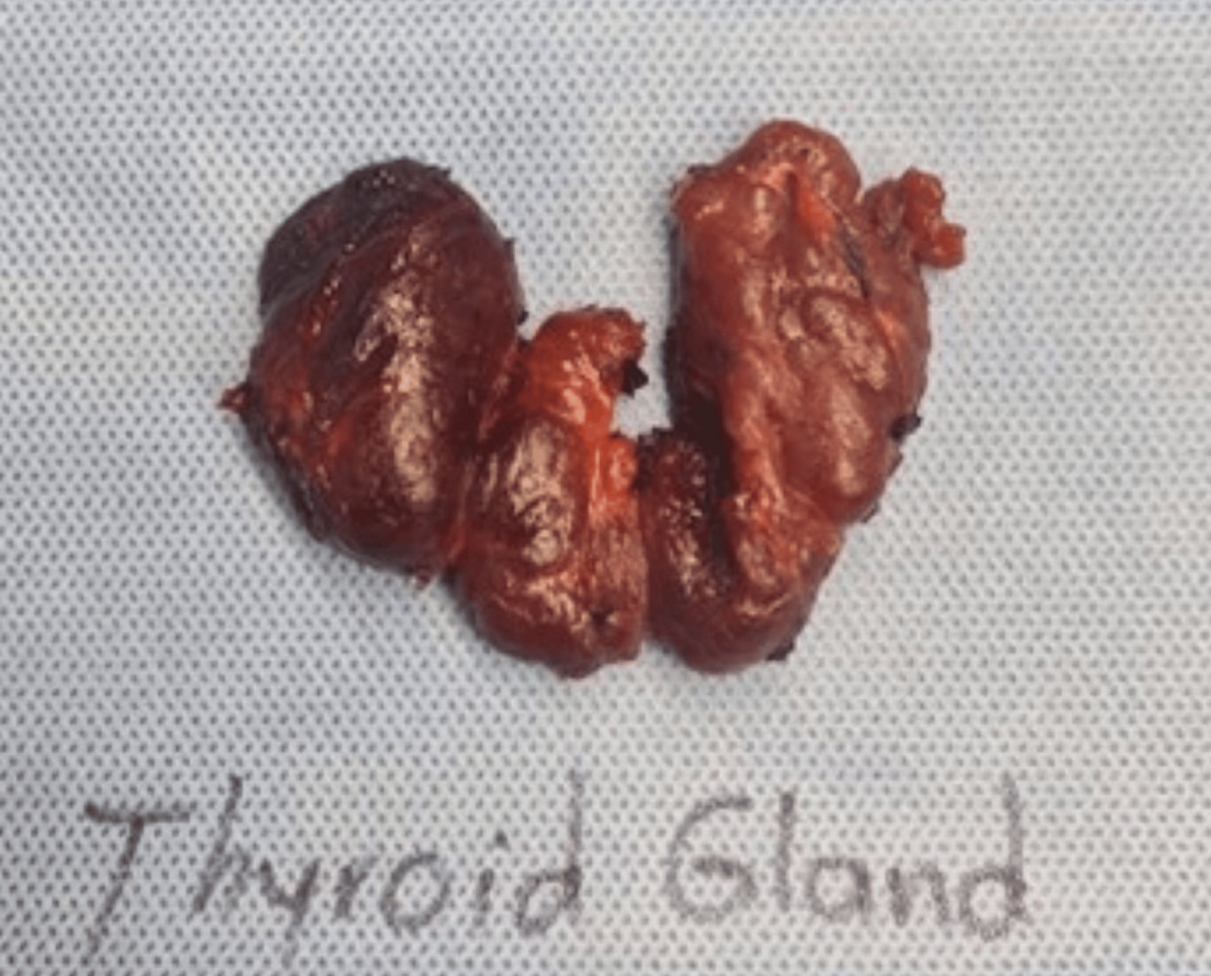
A postoperative image of the thyroid gland specimen following total thyroidectomy shows the complete removal of the thyroid gland, including both lobes and the isthmus.

**Figure 5:**
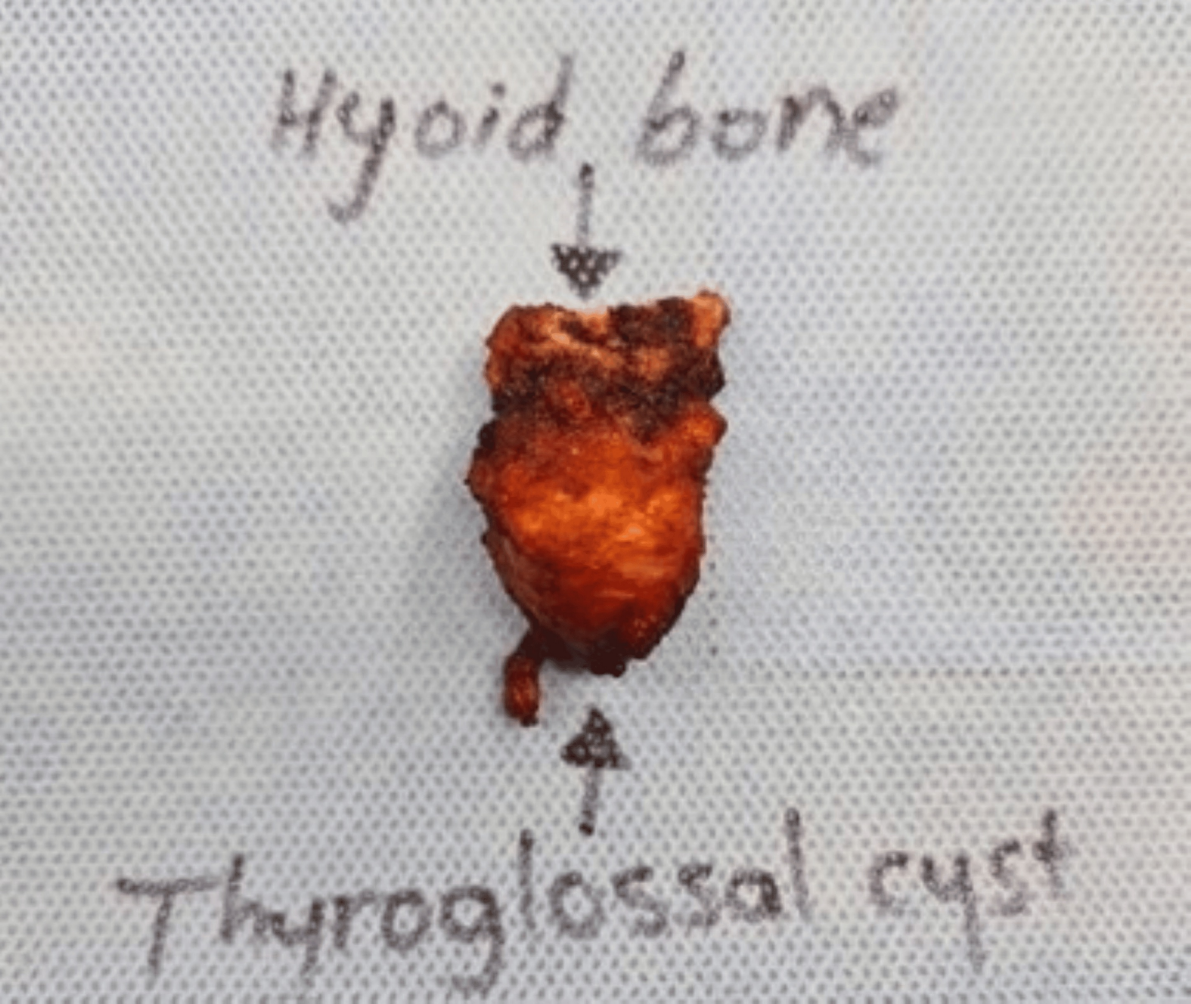
A postoperative image of the thyroglossal duct cyst specimen, along with the central segment of the hyoid bone, after the Sistrunk procedure.

Ultrasound-guided fine needle aspiration cytology was performed on the patient, which confirmed a diagnosis of papillary thyroid cancer. After discussing various treatment options, along with the benefits and potential complications of surgery, the patient consented to undergo a total thyroidectomy combined with the Sistrunk procedure to remove the thyroglossal duct cyst associated with the papillary thyroid cancer. Following the surgery, both the thyroid gland and the thyroglossal duct cyst were sent for histopathological examination (Figures 4, 5).

The patient was doing well in the initial days following surgery, with a clean wound and intact recurrent laryngeal and superior laryngeal nerves, as well as adjacent vasculature, showing no complications. He remained in stable condition. The histopathology report of the samples revealed thyroglossal duct cyst papillary thyroid carcinoma of classic type that is 1.9 cm in its greatest dimension microscopically and unifocal papillary thyroid carcinoma in the left lobe of the thyroid gland that is 0.1 cm in greatest dimension microscopically. Later on, he was referred to medical oncology for more comprehensive care. I-131 radioactive iodine and thyroid suppression therapy were provided after the surgery. The patient has been monitored for one year without any signs of metastasis or recurrence. He has maintained routine clinic follow-ups, and the surgical site healed well.

## Discussion

Thyroglossal duct cyst papillary carcinoma is regarded as an uncommon finding, occurring in roughly 1% of thyroglossal duct cyst cases. Among these, papillary thyroid carcinoma is identified in about 85-91% of instances, followed by squamous, follicular, and mixed carcinomas. While its presentation often mimics that of a benign cyst, it may also present with symptoms like dysphagia or hoarseness. Typically, thyroglossal duct cyst carcinoma manifests as a palpable midline neck mass. Concerns for malignancy should arise if the cyst is fixed, hard, irregular, has associated palpable neck lymph nodes, or shows sudden enlargement [6-9]. Malignant transformation of an untreated thyroglossal duct cyst is a rare but significant complication. Diagnosis is predominantly achieved through histopathological examination of the surgically excised specimen. In one study, women were more frequently affected than men. The average age at presentation was 39 years, with a range from 6 to 81 years [7]. In our case, the patient was a 62-year-old male. Malignant tumors of the thyroglossal duct can originate from two sources: squamous cell carcinoma, which arises from metaplastic columnar cells lining the duct, and thyrogenic carcinoma, which develops from thyroid gland remnants present in the duct or cyst [1]. Thyroglossal duct papillary carcinoma comprises approximately 80% of cases, while the remaining 20% are attributed to squamous cell carcinoma, as reported in 200 cases of thyroglossal duct carcinomas [4]. In our case, the histopathology report revealed a thyroglossal duct cyst with papillary thyroid carcinoma, supporting previous findings that papillary thyroid carcinoma is more prevalent than squamous cell carcinoma in these cases. The Sistrunk procedure is the most frequently performed surgical option for treating a thyroglossal duct cyst. In cases of malignancy, additional treatments may include thyroidectomy, radioactive iodine therapy, and thyroid suppression [1]. In our case, a total thyroidectomy was performed in conjunction with the Sistrunk procedure to excise the thyroglossal duct cyst associated with papillary carcinoma. For pure thyroglossal squamous cell carcinoma, no additional treatment beyond the Sistrunk procedure is recommended. In contrast, total thyroidectomy is advised for thyroglossal duct papillary carcinoma, regardless of clinical or radiological evidence of thyroid involvement. If there is invasion through the duct cyst wall, tumors larger than 1 cm, or suspected foci in the thyroid gland, the preferred approach is total thyroidectomy followed by thyroid-stimulating hormone suppression and I-131 ablation [8].

## Conclusions

This current case report of thyroglossal duct cyst papillary carcinoma contributes to the existing literature on this rare tumor. It should always be considered as a differential diagnosis when evaluating a midline neck swelling. Typically, it is diagnosed postoperatively as an incidental finding during histopathological examination. Surgical treatment, primarily via the Sistrunk procedure, may be accompanied by total thyroidectomy in certain cases. Additionally, thyroid-stimulating hormone suppression and I-131 ablation may be necessary, warranting a multimodal approach. Currently, there are no evidence-based guidelines for optimal surgical management or follow-up due to the rarity of this condition. Therefore, a multidisciplinary team approach is essential for providing comprehensive care.
